# 4.3: Upgrades of B5a (non-invasive) core biopsies to invasive disease at final surgery: a retrospective review across the Scottish Breast Screening Programme

**DOI:** 10.1186/bcr3500

**Published:** 2013-11-08

**Authors:** YT Sim, JC Litherland

**Affiliations:** 1 West of Scotland Breast Screening Centre, Glasgow, UK; 2 Scottish Breast Screening Programme, UK

## Introduction

Women with B5a (non-invasive) preoperative core biopsies upgraded to invasive disease at surgery have a high chance of needing further surgery. The average B5a upgrade rate across UK breast screening programmes is around 20%. Through this Scottish review, we aim to identify factors affecting upgrade rates and ways to improve our performance.

## Methods

This was a retrospective analysis of 1,252 cases of B5a biopsies from the Scottish Breast Screening Programme between 2004 and 2012. Final surgical pathology was correlated with radiological and biopsy factors.

## Results

B5a upgrade rates for the units ranged from 19.2 to 29.2%, with average of 23.6%. Mean sizes of invasive tumours were small (3 to 11 mm). Upgrade rate was significantly higher for cases where the main mammographic abnormality was mass, distortion or asymmetry, compared with microcalcification alone (33.2% vs. 21.7%) (*P *= 0.0004). The upgrade rate was significantly lower with use of large-volume vacuum-assisted biopsy (VAB) devices than 14-gauge core needles (19.9% vs. 26%) (*P *= 0.013). The upgrade rate was lower in stereotactic than ultrasound-guided biopsies (21.2% vs. 36.1%) (*P *< 0.001).

Heterogeneity of data from different units limited evaluation of other potential factors.

## Conclusion

There is variation in practice across Scottish units, including first-line biopsy technique and/or device and protocols for repeat biopsy. Upgrade rates are lower for cases with microcalcification as the sole mammographic feature, and with use of VAB devices. Nevertheless, it is of interest that a few centres maintain low upgrade rates despite not routinely using VAB as the first-line technique for biopsy of microcalcification.

